# eSTGt: a programming and simulation environment for population dynamics

**DOI:** 10.1186/s12859-016-1004-y

**Published:** 2016-04-27

**Authors:** Adam Spiro, Ehud Shapiro

**Affiliations:** Department of Computer Science and Applied Mathematics and Department of Biological Chemistry, Weizmann Institute of Science, Rehovot, Israel

**Keywords:** Stochastic simulation, Population dynamics, Lineage trees, Developmental modeling

## Abstract

**Background:**

We have previously presented a formal language for describing population dynamics based on environment-dependent Stochastic Tree Grammars (eSTG). The language captures in broad terms the effect of the changing environment while abstracting away details on interaction among individuals. An eSTG program consists of a set of stochastic tree grammar transition rules that are context-free. Transition rule probabilities and rates, however, can depend on global parameters such as population size, generation count and elapsed time. In addition, each individual may have an internal state, which can change during transitions.

**Results:**

This paper presents eSTGt (eSTG tool), an eSTG programming and simulation environment. When executing a program, the tool generates the corresponding lineage trees as well as the internal states values, which can then be analyzed either through the tool’s GUI or using MATLAB’s command-line environment.

**Conclusions:**

The presented tool allows researchers to use existing biological knowledge in order to model the dynamics of a developmental process and analyze its behavior throughout the historical events. Simulated lineage trees can be used to validate various hypotheses *in silico* and to predict the behavior of dynamical systems under various conditions. Written under MATLAB environment, the tool also enables to easily integrate the output data within the user’s downstream analysis.

## Background

In recent years there has been a great interest in modeling and simulating various aspects of population dynamics in biological and ecological systems [1–4]. The increasing computational resources along with a deeper understanding of biological and ecological phenomena have led to the development of many languages for describing, analyzing and simulating concurrent stochastic processes. Many such languages specify Markovian dynamics and differ by level of abstraction, ease and complexity of the description and execution efficiency [5]. Many tools have been developed in order to allow and simplify the use of mathematical modeling for the life-science community, and each one has its strengths and weaknesses [6–9]. There is no single tool that has all the required features and choosing the appropriate one depends on the specific goals and resources of the user. Formalisms such as Chemical Reaction Networks [10] and stochastic Process Algebras [11] have a great descriptive power but are often too complex for the average user. We have previously developed and formulated a simpler and more practical language for modeling and simulating the behavior and interaction of populations [12]. We did so by extending the notion of Stochastic Tree Grammar (STG) [13] by incorporating environment-dependent rates and probabilities to the transition rules. These can be dynamically defined as functions of the system’s state, which include global values such as current population size, generation count or elapsed time. Introducing both rates and probabilities to the transition rules enables a more intuitive and flexible description of biological phenomena and in many cases it fits well to the way biologists think and observe different dynamics. For example, a scenario where the rate of reproduction stays constant but the probability of generating different species changes can be easily described using eSTG. In addition, we extended the language by allowing each individual to have an internal state that can change via transition rules. Here we present eSTGt, a programming and simulation environment for eSTG. A prominent feature of the tool is that it can stochastically produce lineage trees, each corresponding to a different stochastic program execution. These lineage trees record the entire execution history of the process, including the dynamics that led to existing as well as to extinct individuals. Unlike previous systems that produce only population size dynamics [14–17], our tool also outputs the corresponding lineage trees, which can be used to analyze the evolutionary and developmental history of the process.

## Implementation

eSTGt was developed using MATLAB R2013a (The MathWorks, Inc., Natick, MA, USA) and it can be executed either as a GUI program or through MATLAB’s command-line, allowing easier batch processing and parallelization. If needed, the tool can automatically perform multiple executions of a program with different random seeds, producing a stochastic sample of instances from the space of possible outcomes. Written as an open-source program under MATLAB environment, the tool also enables to easily integrate the output data within the user’s downstream analysis.

### Program definition

The program definition is encoded using an XML file along with accompanied MATLAB functions. The XML file encodes the transition rules along with the species names, initial rates and probabilities, initial population size, internal states names and initial values, simulation time, random seed, and conditional transitions, as explained below. The XML text also encodes the names of the accompanied MATLAB functions, which consist of the global updating functions of the rates and probabilities as well as the updating functions of the internal states. XML is a widely used format [4, 8] that enables a succinct and human-readable description and also allows easy editing, parsing and future extensions. The use of MATLAB code for writing the updating functions enables a simple and expressive way to describe the dependency of the global parameters on the system’s state.

An eSTGt modeling and simulation experiment may be based on previous experimental observations. These can be formulated into transition rules and estimated parameters, including transition rates and events probabilities and how they depend on global values (such as population size and time). The model can then be simulated and the results can be used both for model validation and predictions. Validation involves testing the *in silico* reproducibility of experimental observations and a validated model can be used to predict the behavior of the system under new conditions that have not been yet performed experimentally. These predictions can then be experimentally validated and the results can be again used to validate or adjust the model.

### Stochastic simulation

An eSTG transition rule has the following general form [12]:

$$ A\overset{r}{\to }{\left\{{S}_1\right\}}_{p_1}\left|{\left\{{S}_2\right\}}_{p_2}\right|\dots \left|{\left\{{S}_{n-1}\right\}}_{p_{n-1}}\right|{\left\{{S}_n\right\}}_{p_n} $$

where *r* is the transition rate, *p*_*i*_ are the transition probabilities such that ∑_*i* = 1_^*n*^*p*_*i*_ = 1, and each *S*_*i*_ is either an empty group (indicating termination) or a group of either one or two species that are the targets of the transition. A program can have multiple transition rules and the rates and probabilities can depend on the system’s state. Each species contains at most one transition rule in which it occurs on the left side of the transition but can occur on the right side of the different transitions as many times as needed. Each species can also include internal states, which can be updated and inherited through transition execution.

An eSTG program can be stochastically simulated using the Gillespie algorithm [18]. To do so, each eSTG transition rule of the form above is translated into *n* chemical reaction rules [12]:

$$ A\overset{\kern1em {c}_i\kern1.25em }{\to }{S}_i,\kern0.5em {c}_i=r\cdot {p}_i,\kern0.5em i=1..n $$

where *c*_*i*_ is the reaction rate. The propensity functions are then calculated by taking the product of the population reaction rate with the size of the corresponding population. In our implementation we used the Gillespie’s Direct Method algorithm in order to calculate both the time to the next reaction and the reaction’s identity.

### eSTG program examples

The following examples depict the usage of the different eSTGt features, including regulated interaction between different species, the use of internal states and conditional transitions. The examples include the input using an XML format and the corresponding MATLAB files that describe the updating functions.

### Prey/Predator

The prey/predator model of Lotka-Volterra [19] is usually defined using the following ODEs:

$$ \frac{dPrey}{dt}= Prey\left({c}_1-{c}_2 Predator\right) $$

$$ \frac{dPredator}{dt}=- Predator\left({c}_3-{c}_2 Prey\right) $$

These ODEs can be translated into the following eSTGs [12]:

$$ Prey\overset{r_1}{\to }{\left\{ Prey, Prey\right\}}_{p_1}\Big|{\left\{\phi \right\}}_{ow} $$

$$ Predator\overset{r_2}{\to }{\left\{ Predator, Predator\right\}}_{p_2}\Big|{\left\{\phi \right\}}_{ow} $$

with the following updating of the rates and probabilities:

$$ {r}_1={c}_1+{c}_2\cdot \left| Predator\right| $$

$$ {r}_2={c}_2\cdot \left| Prey\right|+{c}_3 $$

$$ {p}_1=\frac{c_1}{r_1} $$

$$ {p}_2=\frac{c_2\cdot \left| Prey\right|}{r_2} $$

These rules are encoded using the following XML and MATLAB code:

The **ExecParams** XML element consists of specific execution parameters such as the simulation time and the random seed. The **FunHandleName** element consists of a handle to a MATLAB function that encodes the global updating of the rates and probabilities as function of the system’s state (see below). Each species is described using a **Rule** element that defines the transition rule along with initial values of the rate, probabilities and initial population size. In the above example there are two transition rules for each of the species with simulation time of 10 units and initial population size of 900 for both the Prey and the Predator. We note, that the initial indicated values of the rates and probabilities (**1** and **0.5** respectively in the **prod** XML element) are arbitrary since they are updated to their appropriate values immediately upon the first transition execution (see the updating function below). The MATLAB code for the updating function **updating_LotkaVolterra** is defined as follows:

The updating function updates the rates and the probabilities according to the definition using specific values for *c*_1_, *c*_2_, *c*_3_.

### Internal states

In this example we simulate stem cell differentiation. *SC* (stem cells) divide symmetrically with rate 0.1, while self-renewing or differentiating with the same probability (50 %), and *Diff* (differentiated cells) can either proliferate (with probability 49 %) or die (with probability 51 %) at rate 1.

We define two internal states called *MS*, which simulates somatic mutations of Microsatellites (MS) [20] and *Gen*, which counts the number of generations since each differentiation.

*MS Internal state*: We define a vector of *n* variables $\overrightarrow{MS}=\left(M{S}_1,\dots M{S}_n\right)$, which correspond to the number of repeats in *n* MS loci in the DNA. In every cell division, the number of MS repeats for each locus changes according to the stochastic function *f*_*MS*_, which can cause either a decrease or an increase of one repeat with probability *p* [21]:

$$ {f}_{MS}(x)=\left\{\begin{array}{l}\begin{array}{l}x+1\  with\  probability{\scriptscriptstyle \frac{p}{2}}\hfill \\ {}x-1\  with\  probability{\scriptscriptstyle \frac{p}{2}}\hfill \end{array}\hfill \\ {}x\  otherwise\hfill \end{array}\right. $$

We define the following transition rules:

$$ \begin{array}{l}SC\left(\overrightarrow{MS}={\overrightarrow{x}}_{MS}\right)\overset{0.1\ }{\to }{\left\{SC\left(\overrightarrow{MS}={f}_{MS}\left({\overrightarrow{x}}_{MS}\right)\right),SC\left(\overrightarrow{MS}={f}_{MS}\left({\overrightarrow{x}}_{MS}\right)\right)\right\}}_{0.5}\Big|\\ {}\kern0.16em {\left\{ Diff\left(\overrightarrow{MS}={f}_{MS}\left({\overrightarrow{x}}_{MS}\right)\right), Diff\Big(\left(\overrightarrow{MS}={f}_{MS}\left({\overrightarrow{x}}_{MS}\right)\right)\right\}}_{0.5}\\ {} Diff\left(\overrightarrow{MS}={\overrightarrow{x}}_{MS}\right)\overset{1}{\to }{\left\{ Diff\left(\overrightarrow{MS}={f}_{MS}\left({\overrightarrow{x}}_{MS}\right)\right), Diff\left(\overrightarrow{MS}={f}_{MS}\left({\overrightarrow{x}}_{MS}\right)\right)\right\}}_{0.49}\Big|{\left\{\phi \right\}}_{0.51}\end{array} $$

This model can be used for example to simulate cell lineage reconstruction using MS somatic mutations [22].

*Gen internal state*: The following transition rules include the internal state *Gen*, which counts the number of generations since each differentiation event:

$$ SC\overset{0.1}{\to }{\left\{SC,SC\right\}}_{0.5}\Big|{\left\{ Diff\left(Gen=1\right), Diff\left(Gen=1\right)\right\}}_{0.5} $$

$$ Diff\left(Gen=x\right)\overset{1}{\to }{\left\{ Diff\left(Gen=x+1\right), Diff\left(Gen=x+1\right)\right\}}_{0.49}\Big|{\left\{\phi \right\}}_{0.51} $$

The following XML represents the above transition rules and internal states (the symbol **ow** stands for *otherwise* and equals one minus the sum of the other probabilities):

The **InternalState** element includes the internal state’s names, initial value, updating function name and duplication number, which indicates how many instances of that internal state are simulated. Note, that in this example the rates and probabilities are not updated and so the updating function is empty:

However, we now have to define an updating function for the internal states *MS* and *Gen*, namely:

The input of an internal state updating function is the current value of the internal state and the output is the updated value.

### Conditional transitions

Conditional transitions enable to transform each individual instance into another species or to termination (death) if a certain condition on its internal states is met. Each individual instance is examined upon each transition event and if its internal state follows the defined condition that individual is transformed to the defined target.

The following toy example shows three species, two types of “stem-cells” where one divides symmetrically and another one divides asymmetrically and a differentiated cell, which divides symmetrically or die. The asymmetric stem cells and the differentiated cells contain an internal state counter, which increases its value stochastically. The asymmetric stem cell includes a conditional transition that causes it to transform into a differentiated cell when the counter reaches a certain threshold, and the differentiated cells include a conditional transition that causes it to die when the counter reaches a second threshold. This is described by the following rules:

$$ SCASym\overset{0.1}{\to }{\left\{ SCASym, SCSym\right\}}_1 $$

$$ SCSym(CounterStoch)\overset{0.1}{\to }{\left\{ SCSym, SCSym\right\}}_1 $$

$$ Diff(CounterStoch)\overset{1}{\to }{\left\{ Diff, Diff\right\}}_{0.5}\Big|{\left\{\phi \right\}}_{0.5} $$

with the internal state updating:

$$ CounterStoch= CounterStoch+ normrnd\left(1,0.1\right) $$

and the conditional transitions:

$$ \begin{array}{cc}\kern1em  SCSym\overset{\left( CounterStoch>5\right)}{\to } Diff\kern1em & \kern2em \\ {} Diff\overset{\left( CounterStoch>10\right)}{\to}\phi \kern1em \end{array} $$

where *normrnd*(1, 0.1) is a random sampling from a normal distribution with mean 1 and std of 0.1.

The following XML represents the above transition rules, internal states and conditional transitions:

The structure of the conditional transition is such that the **ConditionalTransition** element includes any condition on the internal states of that species using MATLAB code syntax and the **Transition** element includes the name of the target species (to which the species is transformed into) or **{0}** for termination (death).

The updating function of the internal state **CounterStoch** is as follows:

The **CounterStoch** internal state increases stochastically each time a transition event is executed.

## Results and discussion

In our previous paper [12] we presented the usability of eSTG by presenting a variety of examples that can be modeled and simulated using this approach, including complex stem-cell dynamics, different strategies for feedback regulation, prey/predator, Luria-Delbrück, accumulation of somatic mutations and others. The simulation results of these scenarios were all defined and executed using eSTGt, and the corresponding code for these programs and the programs described in this paper can be found in the project’s homepage (see Availability and requirements Section).

**Fig. 1:**
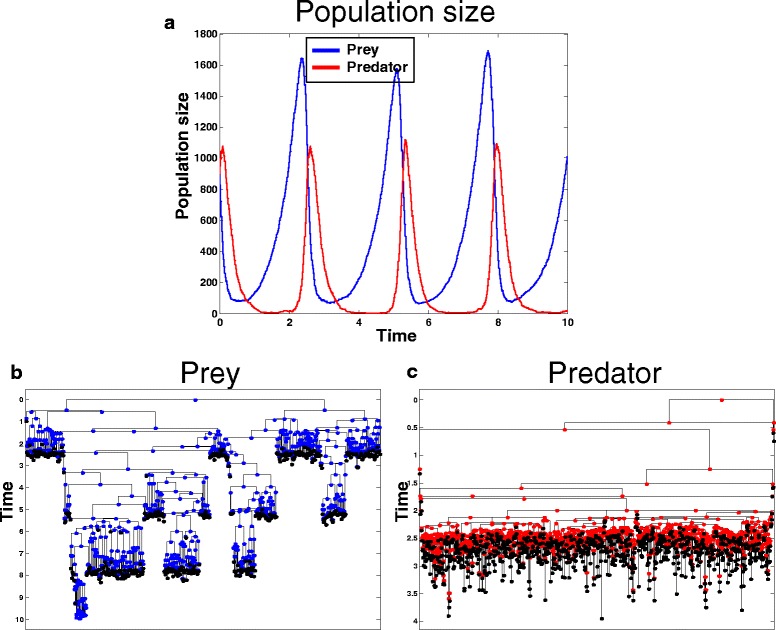
An output example of the Lotka-Volterra program execution. An output example of the executed program described in the main text (adapted from [12]). **a** Population size as a function of time. **b** A lineage tree of one of the 900 originating Preys. **c** A lineage tree of one of the 900 originating Predators. Both (b) and (c) exhibit the characteristic bottleneck phenomenon, where most lineages get extinct

For example, Fig. 1 shows the results of an example execution of the Prey/Predator program depicted before. Figure 1a shows the characteristic population size dynamics as a function of time and Figs. 1b,c show example lineage trees originated from one of the originating species (prey and predator respectively). The lineage tree in Fig. 1b visually reveals an interesting bottleneck phenomenon where most sub-lineages get extinct during the stage of population size decrease and only a single sub-lineage survives. This sub-lineage corresponds to a sub clone of the population, which can explain events such as genetic drift or fixation.

### Use case example: *In silico* assessment of phylogenetic reconstruction algorithms

**Fig. 2:**
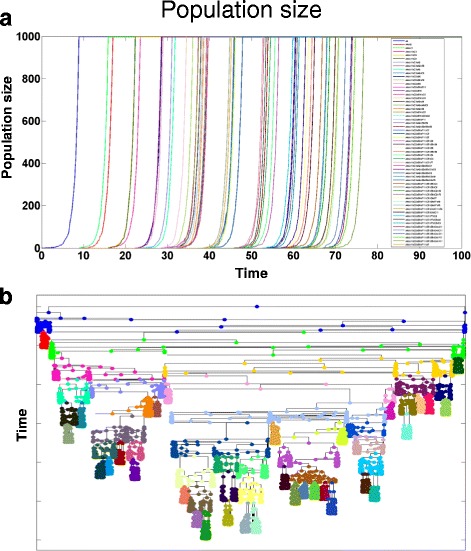
Results of the *ex vivo* simulation. Simulation result of the *ex vivo* scenario. Each clone consists of 1000 single cells from which several single cells are selected to initiate new clones. Total of 58 clones were generated from 9 different seeding time points. **a** Population size dynamics of the simulated tree. Once a clone reaches the size of 1000 several single cells are selected to initiate new clones and the other cells stop dividing. **b** The resulted cell lineage tree on which the accuracy of reconstruction algorithms is examined

A prominent feature of eSTGt is its inherent ability to generate lineage trees that capture the entire evolutionary dynamics from the earliest ancestor down to the extant and extinct individuals. This makes it a very convenient tool for the analysis of phylogenetic trees, and specifically for *in silico* evaluation of tree reconstruction accuracy using genomic data. As we showed, eSTGt can be conveniently used to simulate different scenarios of evolutionary phylogenetics including the modeling of genomic mutations, which accumulate through divisions. Extracting this mutational data from the leaves of the tree (corresponding to extant individuals) and feeding it into tree reconstruction algorithms enables to easily evaluate the tree reconstruction accuracy by comparing the reconstructed tree to the real tree by using one of the many methods for phylogenetic trees comparison [23]. In our lab we conducted an experiment where we generated an *ex vivo* cell lineage tree by repeatedly sampling single cells that went through clonal expansion. This process generated an *ex vivo* cell lineage tree with a known topology in which each single-cell clone is represented by a node in the tree. We then sampled single cells from each clone and sequenced their DNA in order to discover somatic mutations. These mutations were used to evaluate the tree reconstruction accuracy by comparing the reconstructed tree with the true one. Using eSTGt we simulated the *ex vivo* experiment along with the somatic mutations and analyzed the results in order to validate the experimental data and to predict the impact of different mutation rates and future single-cell genotyping enhancements on the tree reconstruction accuracy (manuscript submitted). Figure 2 shows the result of a simulated lineage tree. The full eSTG program can be found in the project’s homepage.

### The GUI interface

**Fig. 3:**
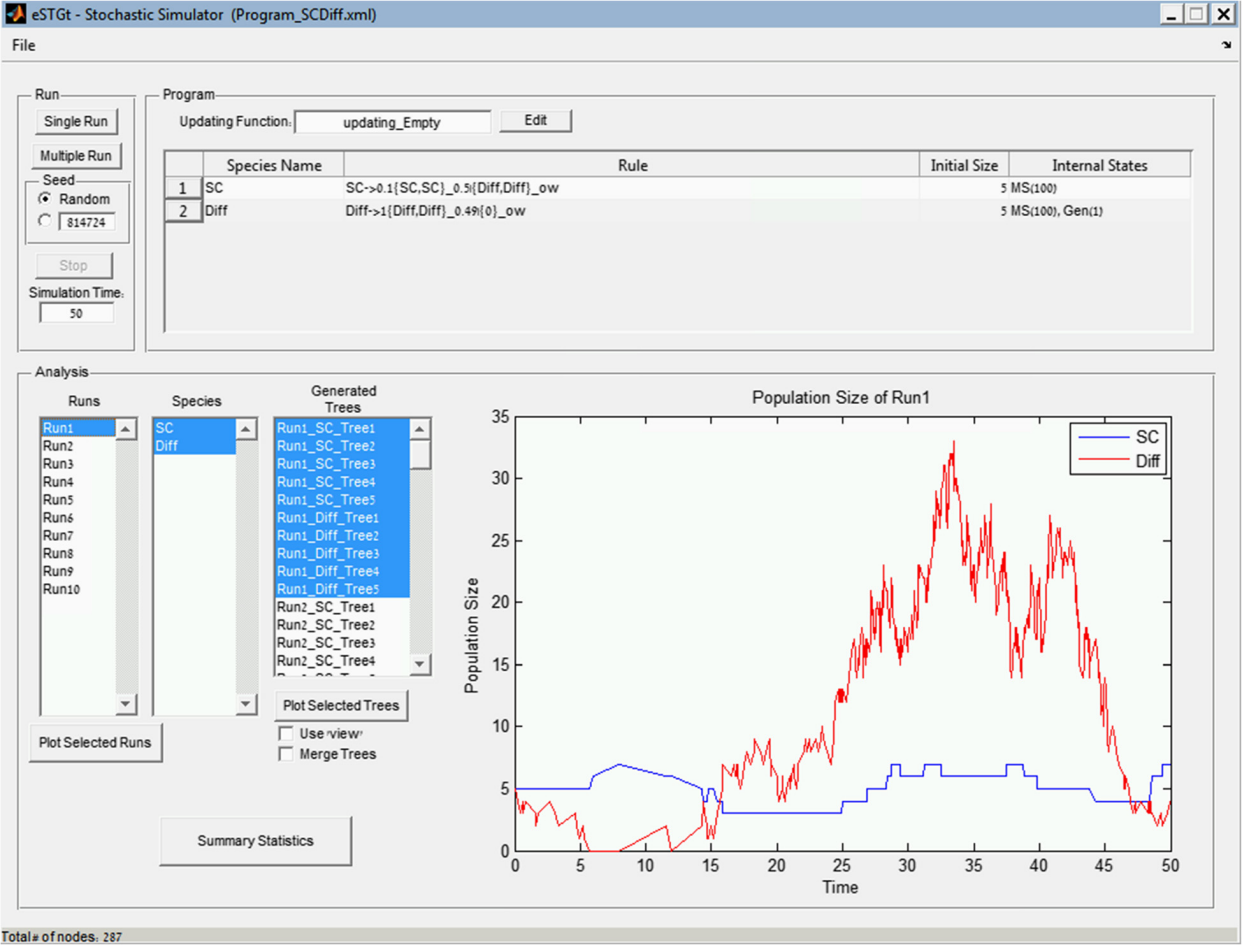
The main window of the GUI. The window is divided into 3 panels, namely “Program”, “Run” and “Analysis”. The “Program” panel includes the transition rules and the internal states details as parsed from the input XML file. The “Run” panel enables to execute a single or multiple simulations using different random seeds and set the simulation run time. The “Analysis” panel includes the output of the executions. For each run the corresponding population size graph is presented and the generated lineage trees can be displayed and analyzed

The main window of the GUI interface is presented in Fig. 3. The GUI enables to load an eSTG program, run it using various random seeds and analyze the results.

After loading an XML file the program details are presented. The GUI allows to execute either a single simulation or a batch of consecutive simulations using different random seeds. Each simulation is then displayed separately in a list box, which allows the user to select single or multiple simulation results for further analysis. Each initiating species acts as a root of a lineage tree, which can be visualized by selecting the tree from the list box of generated trees and pressing the “Plot Selected Trees” button. It is also possible to merge multiple trees by marking the appropriate check box.

The GUI also displays and allows editing the global updating function, which is written in MATLAB code and can access the population size of all species as well as the current time.

During an execution of a program the population size of all species is displayed in real-time, allowing the user to observe the advancement of the execution. After the execution is completed the details of all the simulations, including the initiating species and the generated trees are displayed. When selecting a specific simulation execution the population size of the selected species is displayed.

The GUI also allows the user to save the current session into a ".mat" file (MATLAB’s binary format for storing workspace variables) for future loading either through the GUI or through regular MATLAB environment for further downstream analysis. It is also possible to save the generated trees into the corresponding text files in Newick format, and the internal states values, which are saved into tab-separated files.

### Summary Statistics

**Fig. 4:**
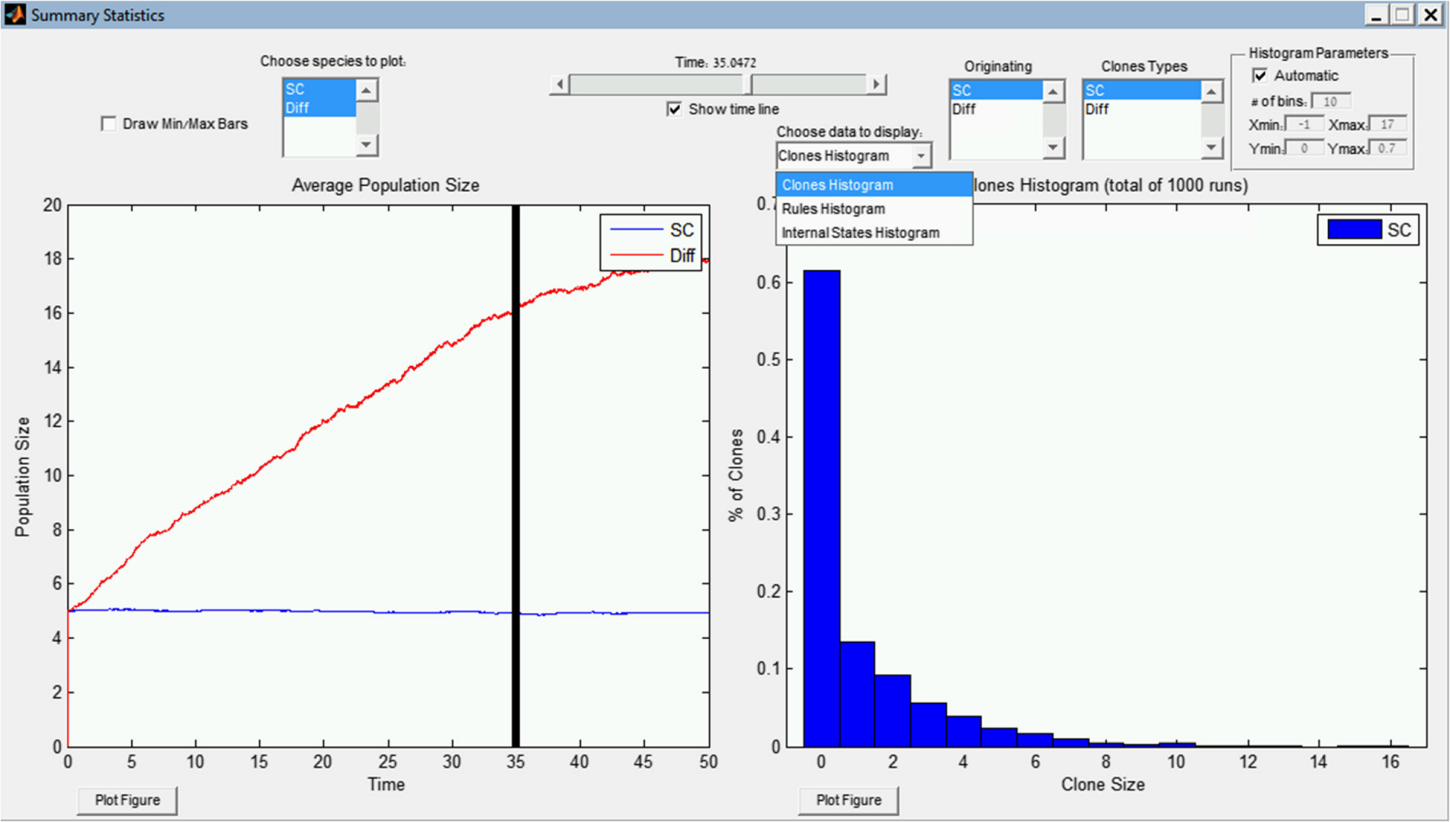
The GUI windows of the Summary Statistics. Summary statistics over all the simulation runs. The presented data is the result of 1000 stochastic simulations of the “Internal states” program described in the main text

The “Summary Statistics” window presents various statistics over all simulation runs and enables to scroll over the simulation time in order to get snapshots of the statistics for each historical time point. Figure 4 shows an example of an output window where the left panel presents the average population size over all simulation runs and the right panel can present three different types of statistics according to the selected option in the dropdown menu. The options are:

“Clones Histogram”: A clone is defined as all individuals that are descendants of a single founder. The clone size is the number of living individuals of that clone. The histogram presents the percentage of clone sizes over all runs. The user can select the originating species types and the species types of the resulting individuals within the clone.

“Rules Histogram”: Displays a histogram of the number of times each rule has been executed over all simulation runs.

“Internal States Histogram”: Displays a histogram of the Internal States values over all runs.

### The command-line interface

The command-line interface enables to run the simulations directly from MATLAB’s command-line. It makes it possible to execute programs without GUI support directly from a Linux system prompt, allowing easy batching and parallelization on a computer cluster. The following commands execute an example program 10 times using different random seeds (example input files are provided as part of the eSTGt source code):

The function **ParseeSTGProgram** receives as input an XML file containing the eSTG program and returns a struct that contains the corresponding rules. This struct is then passed to the second function **RunSim**, which receives as input the rules, an array of random seeds and the simulation time span. The output is a structure array of the runs for each seed and a common data structure for all runs. The output includes the entire data history of the simulations, including events time points, historical population size, executed transition rules, and a struct array that includes detailed information on all the generated nodes, including their creation time, their parents and children relationships, and internal states values. This data enables to easily access the entire historical data, which led to the final system’s state.

## Conclusions

Translating biological knowledge into a well-defined formalism with which one can easily describe, simulate and analyze the system in mind is not an easy task. Formalisms that are too complex can turn this task into a great burden, and simple ones may just not have enough descriptive power. Population dynamics that involve individual interactions can be captured using directed acyclic graphs, which can be extremely complicated, not intuitive, and require enormous computational resources to simulate and analyze. On the other hand, ignoring the interactions between the different species is not realistic. Although the eSTG formalism does not allow direct interaction between different species, we believe that it presents an elegant compromise between a high descriptive power and a simple formalism. Abstracting away individual interactions makes a single rule for each species sufficient and enables the recording of the entire dynamical history of the population using a lineage tree representation. This tree captures all past events and includes, in addition to the living population at every time point, the death of individuals, extinct lineages and historical transition events (e.g. differentiation, symmetrical/asymmetrical divisions).

We believe that eSTGt can contribute to the modeling and simulation approach of developmental dynamics and thus facilitate research in systems biology.

## Availability and requirements

**Project name:** eSTGt

**Project home page:**https://github.com/shapirolab/eSTGt

**Operating system(s):** Platform independent

**Programming language:** MATLAB

**Other requirements:** MATLAB R2013a or higher

**License:** GNU GPL

**Any restrictions to use by non-academics:** None
