# Erratum to: eSTGt: a programming and simulation environment for population dynamics

**DOI:** 10.1186/s12859-016-1155-x

**Published:** 2016-08-10

**Authors:** Adam Spiro, Ehud Shapiro

**Affiliations:** Department of Computer Science and Applied Mathematics and Department of Biological Chemistry, Weizmann Institute of Science, Rehovot, Israel

## Erratum

After publication of the original article [1] it was brought to our attention that important information was missing from the Acknowledgments. The completed Acknowledgments section should read as follows:
